# Loss of *Drosophila* pseudouridine synthase triggers apoptosis-induced proliferation and promotes cell-nonautonomous EMT

**DOI:** 10.1038/cddis.2015.68

**Published:** 2015-03-26

**Authors:** R Vicidomini, A Di Giovanni, A Petrizzo, L F Iannucci, G Benvenuto, A C Nagel, A Preiss, M Furia

**Affiliations:** 1 Dipartimento di Biologia, Università di Napoli 'Federico II', via Cinthia, Naples 80126, Italy; 2 Stazione Zoologica Anton Dohrn, Villa Comunale, Napoli 80121, Italy; 3 Institut für Genetik, Universität Hohenheim, Garbenstrasse 30, Stuttgart 70599, Germany

## Abstract

Many developing tissues display regenerative capability that allows them to compensate cell loss and preserve tissue homeostasis. Because of their remarkable regenerative capability, *Drosophila* wing discs are extensively used for the study of regenerative phenomena. We thus used the developing wing to investigate the role played in tissue homeostasis by the evolutionarily conserved eukaryotic H/ACA small nucleolar ribonucleoprotein pseudouridine synthase. Here we show that localized depletion of this enzyme can act as an endogenous stimulus capable of triggering apoptosis-induced proliferation, and that context-dependent effects are elicited in different sub-populations of the silenced cells. In fact, some cells undergo apoptosis, whereas those surrounding the apoptotic foci, although identically depleted, overproliferate. This overproliferation correlates with ectopic induction of the Wg and JAK-STAT (Janus kinase-signal transducer and activator of transcription) mitogenic pathways. Expression of a p35 transgene, which blocks the complete execution of the death program and generates the so-called ‘undead cells', amplifies the proliferative response. Pseudouridine synthase depletion also causes loss of apicobasal polarity, disruption of adherens cell junctions and ectopic induction of JNK (c-Jun N-terminal kinase) and Mmp1 (matrix metalloproteinase-1) activity, leading to a significant epithelial reorganization. Unexpectedly, cell-nonautonomous effects, such as epithelial mesenchymal transition in the contiguous unsilenced squamous epithelium, are also promoted. Collectively, these data point out that cell–cell communication and long-range signaling can take a relevant role in the response to pseudouridine synthase decline. Considering that all the affected pathways are highly conserved throughout evolution, it is plausible that the response to pseudouridine synthase depletion has been widely preserved. On this account, our results can add new light on the still unexplained tumor predisposition that characterizes X-linked dyskeratosis, the human disease caused by reduced pseudouridine synthase activity.

The control of cell growth and proliferation is a fundamental aspect of tissue homeostasis. To maintain homeostatic conditions, different subsets of cells are continuously required to respond coordinately to external and intrinsic stimuli, to keep the appropriate balance between death, proliferation and differentiation.

The overall capacity of the protein synthetic machinery has an obvious rate-limiting regulatory role in cell growth and division, and production of ribosomes is directly coupled with these processes. Moreover, in a growing number of cases mutations in ribosome components proved to regulate not only the overall translational capacity but also to affect specific developmental or differentiative events, revealing more specialized functions in translational regulation.^1, 2^ Mutations in factors that allow synthesis, processing and modification of rRNA, assembly and nuclear export of preribosomal particles or ribosome translational efficiency also cause tissue- or cell-specific phenotypes and produce a variety of diseases, collectively indicated as ribosomapathies.^3^ Eukaryotic rRNA pseudouridine synthases are among these factors. These ubiquitous nucleolar proteins are conserved from Archaea to man and associate with other conserved core proteins and H/ACA small nucleolar RNAs (snoRNAs) to compose the functional H/ACA snoRNP complexes, whose activity is known to be involved in rRNA processing and site-specific pseudouridylation of rRNA and snRNAs,^4^ as well as of mRNAs and additional classes of noncoding RNAs.^5^

Well-established, rRNA undergoes extensive modifications that influence its processing, folding and functionality. For example, reduction of rRNA pseudouridylation affects ribosome translation fidelity^6^ and modulates the efficiency of internal ribosome entry site-dependent translation,^7, 8, 9, 10^ outlining a crucial role in the regulation of translation specificity. The high biological relevance of rRNA pseudouridine synthases is further testified by the fact that reduced levels or hypomorphic mutations in the human coding gene cause the human X-linked dyskeratosis (X-DC) multisystemic disorder.^11^ Beside H/ACA snoRNPs, the human pseudouridine synthase, called dyskerin, is also a component of the active telomerase complex; this dual role makes it difficult to distinguish between the effects related to loss of snoRNP functions and those caused by telomere attrition. As a consequence, whether X-DC must be regarded primarily as a ribosomopathy or as a telomere disease is still being debated. Considering the availability of sophisticated genetic tools, *Drosophila melanogaster* can represent an advantageous model organism to dissect the multiple roles played by pseudouridine synthases. *Drosophila* dyskerin is 66% identical and 79% similar to human dyskerin, and is equally involved in rRNA processing and pseudouridylation;^12^ however, it has no established role in the maintenance of telomere integrity, as fly telomeres are maintained by insertion of specific retrotransposons at chromosome ends.^13^ This divergent procedure of telomere maintenance makes *Drosophila* an ideal organism to delineate the range of biological effects specifically triggered by loss of H/ACA snoRNP activity, especially focusing at the interface among cell growth, proliferation and differentiation. Because of their striking capacity of undergoing regenerative growth,^14^ the imaginal wing disc provides an ideal system in which to investigate how growth, proliferation and differentiation are coordinated in the context of a developing organ. In fact, cell proliferation is essentially uniform across the whole disc^15^ and is controlled by the same morphogens that govern wing patterning. Gradients of two morphogens, decapentaplegic (Dpp), a member of the transforming growth factor-*β* family, and wingless (Wg), a member of the Wnt family, establish a tight link between cell proliferation and developmental programs. During disc development, a gradient of Dpp determines the anterior/posterior (A/P) axis, whereas a gradient of Wg subsequently defines the dorsal/ventral (D/V) axis, giving rise to the A/P and D/V compartments.^16^ We previously showed that localized depletion of *Drosophila* dyskerin in the wing discs not only caused cell death but also additionally triggered a variety of developmental defects.^17^ Here, we further investigated the role of *Drosophila* dyskerin in wing disc homeostasis and demonstrate that its loss can induce regenerative growth and extensive tissue remodeling, as well as stimulate cell-nonautonomous events of cell fate changes that result in epithelial–mesenchymal transition (EMT).

## Results

### Effect of *mfl* silencing on wing growth and patterning

**Figure 1:**
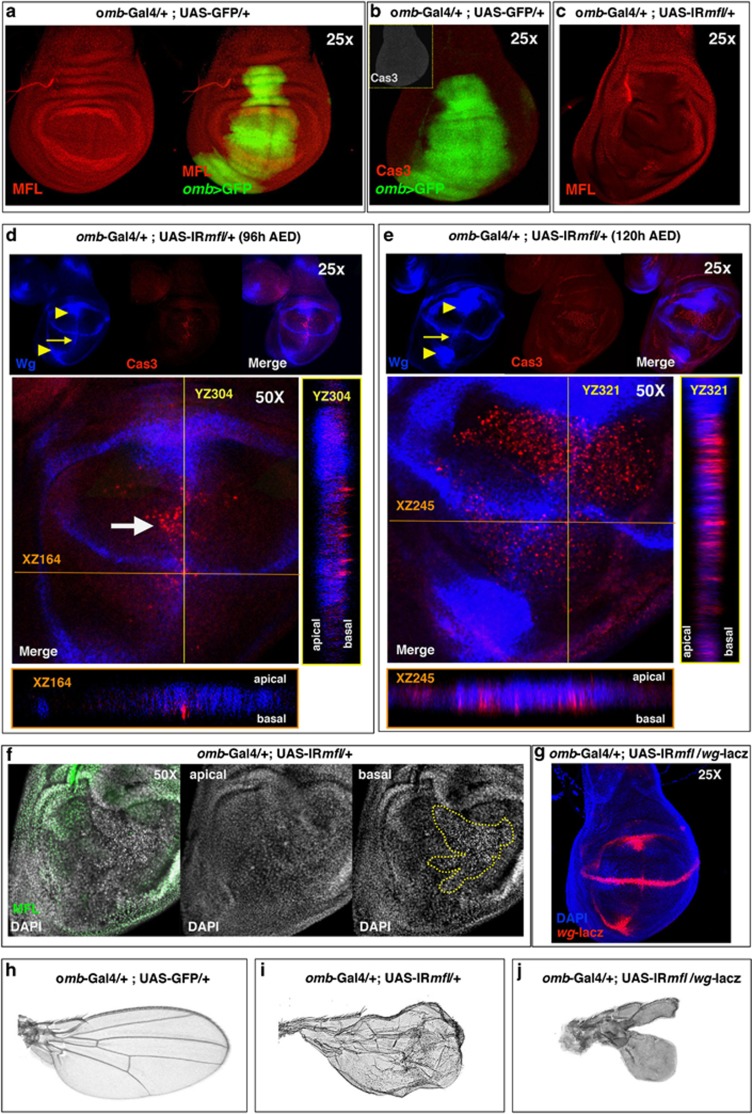
Mfl depletion induces both Cas3 and Wg activation in the wing discs. (**a–g**) Confocal analysis of control and *omb*>*IRmfl* silenced wing discs (v46282 line). (**a**) Ubiquitous expression of Mfl protein (in red) in an *omb*>GFP control disc, in which GFP (green) marks the *omb* expression domain. (**b**) Cas3-positive spots are not present in the *omb*>GFP controls. GFP in green and Cas3 in red (gray in the inset). (**c**) An *omb*>*IRmfl* silenced disc showing efficient and localized Mfl depletion within the *omb* domain (Mfl in red). (**d** and **e**) *omb*>*IRmfl* discs collected at 96 h (**d**) and 120 h AED (**e**). At 96 h AED, clusters of Cas3-positive spots mark the A/P boundary (white arrow), flanked by Wg ectopic induction (see yellow arrow at the top); Wg accumulates also at the middle of the inner ring (see yellow arrowheads on the top). At 120 h AED, the number of Cas3-positive spots increased, concomitantly with Wg ectopic induction along the A/P border (see yellow arrow at the top) and Wg upregulation at the inner ring (see yellow arrowheads at the top). Z-stack confocal analysis showed that most Cas3 signals lie basally and do not overlap Wg staining (see XZ and YZ projections). Cas3 in red and Wg in blue. (**f**) DAPI (4',6-diamidino-2-phenylindole) staining of an *omb*>*IRmfl* disc shows basal clusters of pyknotic nuclei within the silenced domain (area encircled by the yellow line); DAPI is in gray and Mfl in green. (**g**) Expression of *wg-lacZ* reporter in *omb*>*IRmfl* silenced discs confirms that *wg* expression is transcriptionally upregulated compared with controls (see Figure 4d) and marks the same areas where Wg protein accumulates (DAPI in blue and *β*-gal in red). (**h–j**) Comparison of an *omb*>GFP adult wing, taken as control (**h**), with an *omb*>*IRmfl* silenced wing, whose blade appears crumpled and highly disorganized (**i**), and an *omb*>*IRmfl;wg-lacZ* wing (**j**), in which Wg haploinsufficiency strongly enhances the growth defects (see also Supplementary Figure 1)

*Drosophila* dyskerin, called Mfl, is encoded by the *Nop60B/minifly* (*mfl*) gene.^12, 18, 19^ To define in more detail how Mfl loss can affect growth and development, we silenced Mfl activity in the area of the wing disc that flanks the A/P border and includes the main organization center of patterning. Gene silencing made use of the Gal4/UAS system^20^ and was directed by the *omb* (optomoter blind)-Gal4 driver line, which is expressed in the wing central domain from between presumptive veins 1 and 2 in the A compartment to presumptive veins 4 and 5 in the P compartment.^21^ Two different UAS-*IRmfl* target lines were used (v46282 and no. 36595); both lines had no predicted off-targets and gave identical results. The v46282 line already proved to knock down efficiently all *mfl* mRNAs.^17^ The effects of *mfl* silencing were then carefully followed within the *omb* domain of silenced discs (Figures 1a–c). Discs were collected at two developmental times during the third larval instar, that is, at 96 and 120 h after egg deposition (AED), and costained with anti-caspase-3 (Cas3) to follow cell death and with anti-Wingless (Wg) to monitor wing patterning and growth. Control discs at 96 h AED did not show any significant Cas3 staining (Figure 1b), whereas clusters of Cas3-positive cells arised along the A/P boundary in the silenced discs (Figure 1d), confirming that cell death is a major consequence of *mfl* downregulation.^17^ However, in the same discs Wg was strongly upregulated at the center of the inner ring, in both D and V compartments, and ectopically expressed along the A/P boundary (Figure 1d). At the later developmental time (120 h AED), the number of Cas3 spots further increased, massively spreading from A/P border throughout the wing pouch. In parallel, Wg accumulation at the inner ring became more conspicuous (Figure 1e). As shown by confocal Z-stack analysis, Wg staining at the D/V border flanked the Cas3 apoptotic signals, most of which were located more basally (Figures 1d and e). The presence of dying cells was further confirmed by a large number of pyknotic nuclei detected basally in the pseudostratified epithelium (Figure 1f). To mark precisely the cells that were actively producing the Wg proliferative signal, we followed the expression of the *wg-lacZ* transcriptional reporter. Activity of the reporter increased in the silenced background, and stained the same areas previously marked by Wg accumulation (Figure 1g). This observation confirmed that the silenced cells surrounding the apoptotic foci were actively producing high level of Wg. Ectopic secretion of Wg at the edge of the dying tissue has been reported by several authors,^22, 23, 24^ and in some cases has been described to be essential for regenerative growth.^24^ Consistent with this view, we noticed that most of the silenced larvae carrying the *wg-lacZ* reporter died at the pupal stage, whereas rare adult escapers all display underdeveloped/deformed wings that miss the central *omb* domain (Figures 1h–j and Supplementary Figure 1). As the *wg-lacZ* allele is mutant owing to the *lacZ* insertion,^25^ silenced larvae carrying this allele have only one active copy of the *wg* gene. With respect to *mfl* silencing alone, which led to disturbed wing morphology with detachment of the two epithelial layers and blistering, the heterozygous *wg* background markedly worsened the silenced phenotype (Figures 1h–j and Supplementary Figure 1). This enhancement is consistent with a dose effect, and suggests that the level of *wg* activation in these silenced flies is inadequate to counteract the developmental defects caused by Mfl depletion.

**Figure 2:**
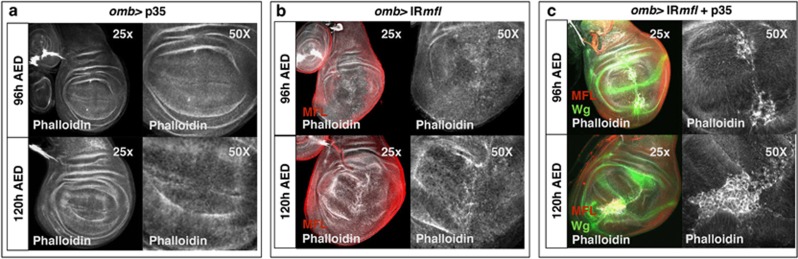
Epithelial remodeling and formation of p35-induced undead cells in the *omb* silenced domain. Confocal analysis of wing discs at 96 and 120 h AED upon phalloidin staining. (**a**) An *omb*>p35 control disc. Phalloidin is in gray. (**b**) An *omb*>*IRmfl* silenced disc (no. 36595 line) exhibiting a strong tissue disorganization. Phalloidin is in gray and Mfl in red. (**c**) Expression of p35 in the silenced discs causes formation of big patches of large undead cells that secret high level of Wg (in green) and cluster along the A/P border

Finally, we analyzed by phalloidin staining the structure of the silenced disc. As shown in Figure 2, the tissue appeared wrinkled, folded and fractured along the A/P border, where clusters of Cas3-positive cells were observed. These local fractures were strongly enhanced in the presence of an UAS transgene that expresses the baculovirus p35 protein, known to inhibit the function of Cas3 but not its activation.^26, 27^ UAS-p35 expression has no effect on the epithelium structure in a wild-type background (Figure 2a), whereas in the silenced discs it generates along the A/P margin patches of large ‘undead cells' that, as typical,^28^ secrete high levels of Wg (Figures 2b and c). The alignment of undead cells along the A/P boundary indicated that these margin cells exhibit a particular sensitivity to Mfl depletion, and first undergo apoptosis. This susceptibility may be an indirect consequence of abnormal formation of the A/P border, possibly due to defects in cell adhesion and/or cell communication.

### *mfl* Silencing elicits context-dependent effects and apoptosis-induced proliferation

A proliferative role has generally been attributed to Wg in regenerative phenomena.^29^ Indeed, we noticed that at both 96 and 120 h AED the silenced discs exhibited morphological alterations in shape, including an evident bending of the A/P boundary that was indicative of localized overgrowth (see Figure 1 and Supplementary Video 1).

**Figure 3:**
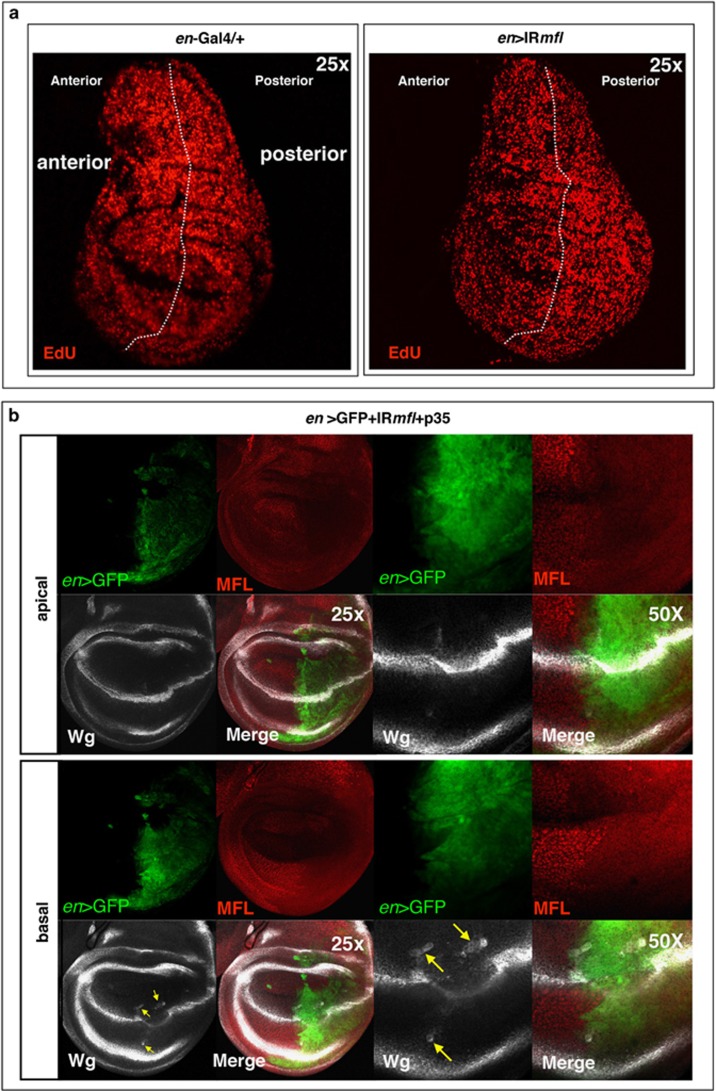
Enhanced proliferation and formation of p35-induced undead cells in the silenced P compartment. Confocal analysis of wing discs at 96 h AED. (**a**) On the left, an *en*/+ control disc, in which EdU labeling marks S-phase cells; on the right, an *en*> *IRmfl* silenced discs (v46282 line) in which EdU labeling stains more intensely the P silenced compartment, indicating that the silenced cells overproliferate. The A compartment serves as internal control. (**b**) Expression of p35 causes formation of undead cells that secrete high level of Wg (in gray) and are dispersed along the irregular A/P border. Note that Wg accumulation at the D/V boundary is detected apically, whereas deformation of the A/P margin is more evident basally, in keeping with previous data^17^

In previous experiments, we triggered *mfl* silencing by the *en*-Gal4 (engrailed-Gal4) driver, whose expression specifically marks the P compartment. Despite the induction of apoptosis, no reduction in the number of phosphohistone H3 (pH3)-positive mitotic cells was noted in the silenced domain.^17^ To further check this aspect, we stained the *en*-Gal4 silenced discs by EdU (5-ethynyl-2′-deoxyuridine) incorporation, to mark with higher sensitivity DNA synthesis and label S-phase cells. This approach highlighted a significant enhancement of the proliferative activity in the silenced P compartment (Figure 3a). According to the previous data,^17^ the A/P boundary was discontinuous and deformed and, upon p35 expression, undead cells that overexpress Wg were detected close to this irregular border (Figure 3b). Note that these cells are located basally, whereas Wg upregulation at the A/P margin is detected only apically.

**Figure 4:**
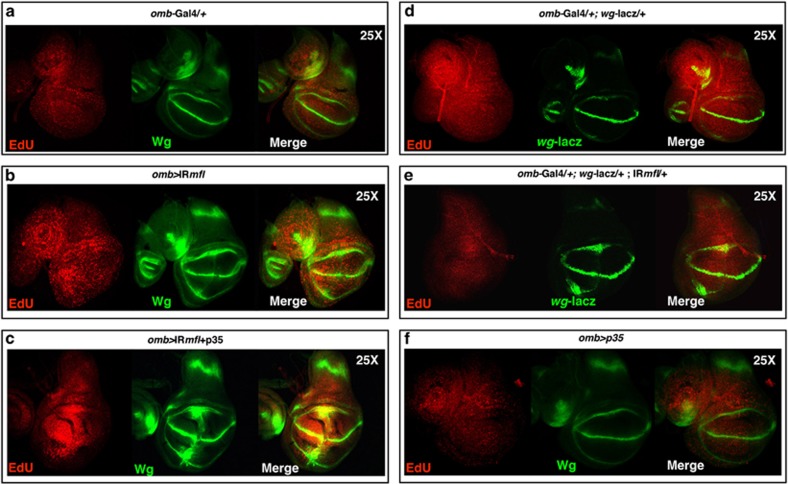
*mfl* Silencing causes apoptosis-induced proliferation. Confocal analysis of wing discs at 96 h AED. (**a**) An *omb/+* control disc labeled by EdU incorporation to mark specifically DNA synthesis and label S-phase cells. (**b**) An *omb*>*IRmfl* silenced disc (no. 36595 line) showing a significant increase of proliferating cells. (**c**) P35 expression further boosts proliferation and elicits a more pronounced hyperplastic overgrowth. EdU is in red and Wg in green. (**d** and **e**) EdU labeling of *omb/+*, *wg-lacZ* (**d**) and *omb*>*IRmfl, wg-lacZ* discs (**e**), both carrying a single active copy of the *wg* gene (*β*-gal is in green). Note that in the *wg-lacZ* background the proliferative activity of the silenced domain is significantly reduced compared with that of *omb*>*IRmfl* discs (**b**). (**f**) Expression of p35 in unsilenced discs has no effect on the proliferation rate (Wg in green)

Enhanced proliferative activity was similarly observed in the *omb* silenced domain. In fact, despite the massive apoptosis (see Figure 1), the overall proliferation rate was not reduced. pH3-positive dividing cells were visualized inside and around Wg-secreting regions, and their number markedly augmented upon p35 expression (Supplementary Figure 2). Consistent with this observation, EdU labeling of S-phase cells was markedly enhanced with respect to controls, and even further by p35 expression (Figures 4a and c; note that in the silenced discs the *omb* domain is expanded and bent). Reliably, upon UAS-p35 expression the rare adult escapers develop wings typified by epithelial refolding, as it occurs upon excessive and hyperplastic overgrowth (Supplementary Figure 3).

**Figure 5:**
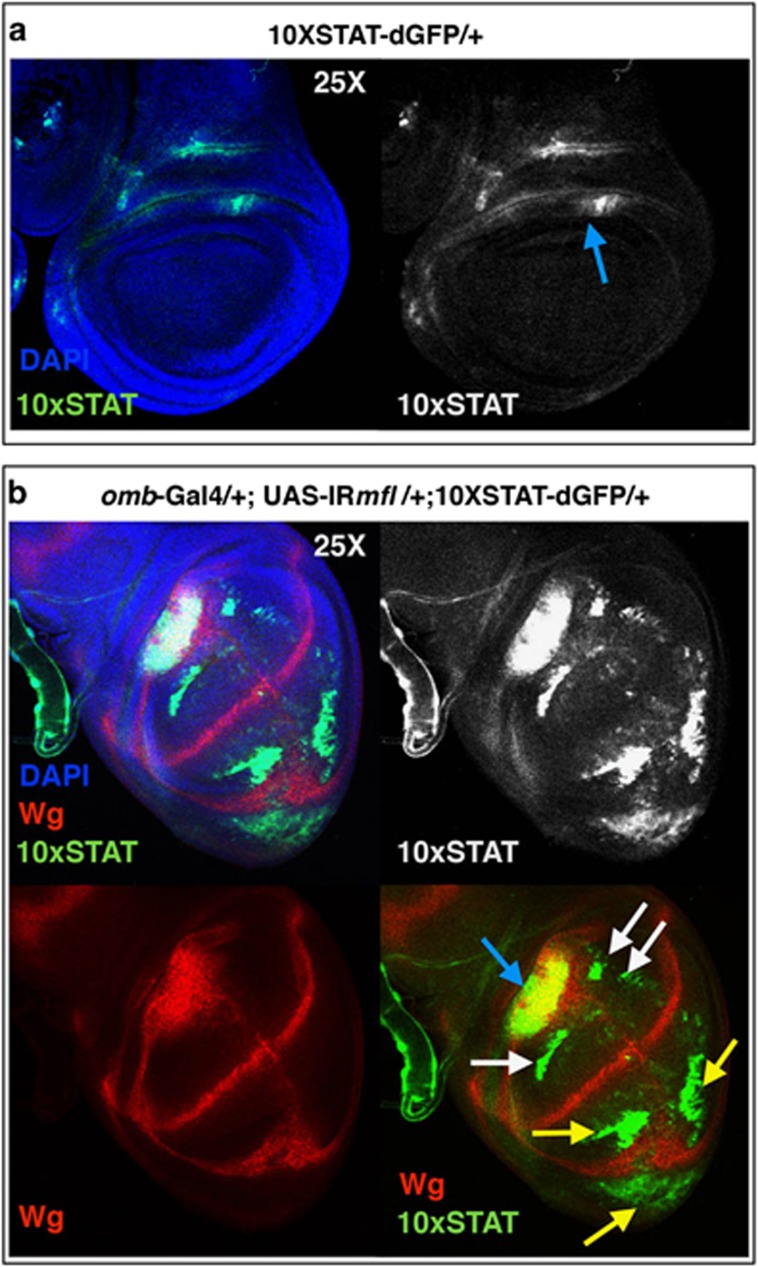
Mfl depletion triggers ectopic activation of JAK-STAT signaling. Confocal analysis of wing discs collected at 120 h AED carrying the 10XSTAT-dGFP reporter (the use of destabilized dGFP^69^ allows to visualize real-time activation of the reporter). (**a**) No activity of the reporter was detectable in the wing pouch of control discs, whereas JAK-STAT expression is detected in the presumptive wing hinge (blue arrow). (**b**) In *omb*>*IRmfl* silenced discs (v46282 line in the picture), the reporter was overexpressed in the wing hinge (blue arrow) and ectopically induced in the dorsal area of the *omb* domain (white arrows), where it, in part, surrounded and overlapped the domain of Wg induction. In the ventral region (yellow arrows), JAK-STAT ectopic induction encircled Wg accumulation. DAPI (4',6-diamidino-2-phenylindole) is in blue, Wg in red and GFP in green

Proliferation of the silenced cells also correlated with upregulation and ectopic induction of JAK-STAT, another proliferative pathway associated with apoptosis-induced proliferation and regeneration.^30, 31^ As shown in Figure 5, this pathway is upregulated at the center of the inner ring, and ectopically activated at specific regions of the silenced domain. In the V compartment, its induction surrounded the areas showing Wg accumulation, whereas in the dorsal part it encircled and in part overlapped Wg ectopic expression. Altogether, these data indicate that Mfl depletion induces a regenerative response, which, as observed in other cases,^32^ in the presence of p35 results in a pronounced hyperplastic phenotype.

Intriguingly, the emerged scenario pointed out that Mfl depletion elicits opposite outcomes in diverse wing territories. Although some cells (those at the A/P boundary first) activate Cas3 and undergo apoptosis, other silenced cells undergo robust proliferation.

### The silenced discs show extensive epithelial remodeling and JNK and Mmp1 ectopic activation

As shown above, the silenced discs appeared crumpled and folded (Figure 1 and Supplementary Video 1), indicating the occurrence of extensive tissue reorganization and remodeling. As regenerative processes require widespread tissue restoration, we followed in detail the expression of two typical markers of epithelial restructuring: the apicobasal distribution of the *Drosophila β*-catenin/Armadillo (Arm) and the levels of polymerized F-actin. Within the wing discs, Arm and F-actin are ubiquitously expressed, but they are both strongly stabilized in two stripes surrounding the D/V boundary.^33, 34^

**Figure 6:**
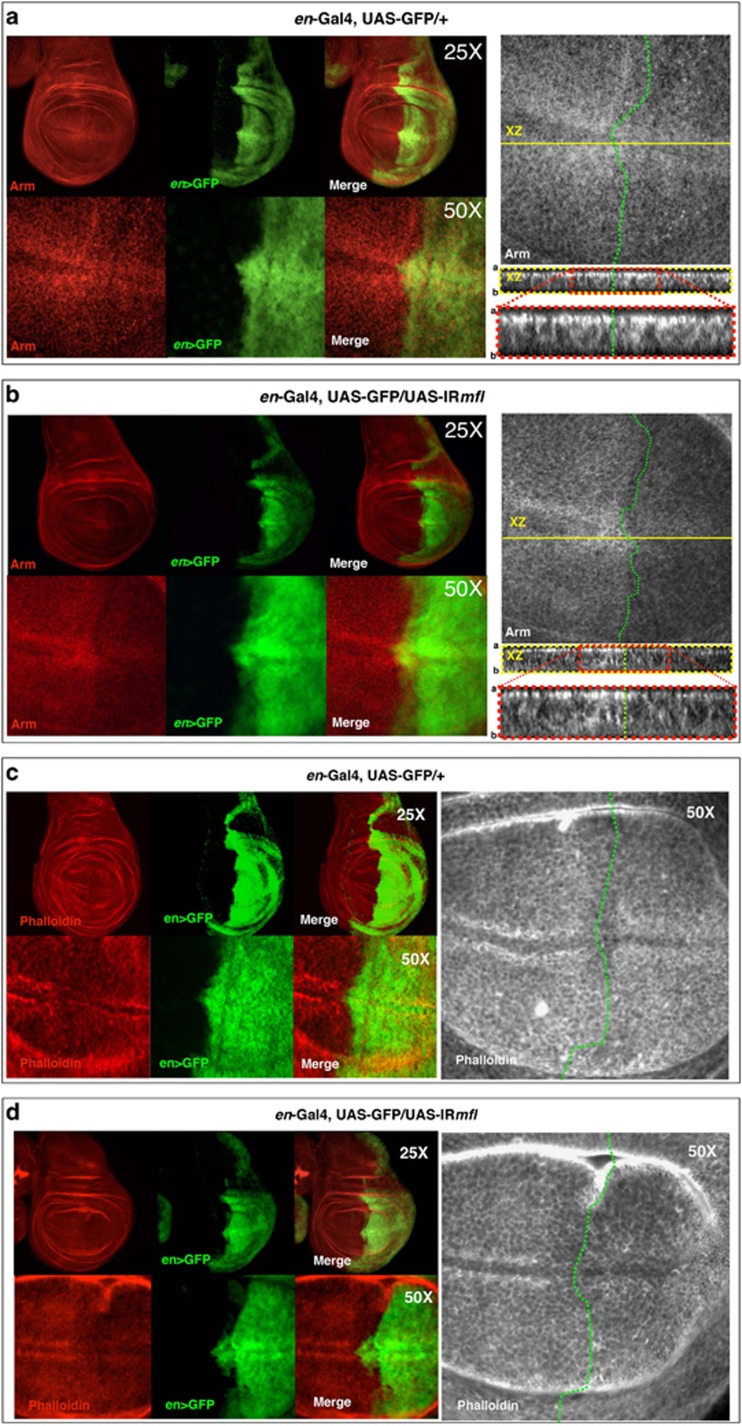
Loss of Arm/*β*-catenin apical localization and reduced F-actin accumulation in the silenced discs. Confocal analysis of wing discs collected at 120 h AED and stained with Arm antibody (in red or gray) or phalloidin (red or gray). (**a**) Localization of Arm protein in *en*>GFP control discs, in which GFP (green) marks the *en* posterior domain. Note that Arm is strongly concentrated in two stripes of cells adjacent to the D/V boundary, and Z-stack analysis (right panels) shows its uniform apical localization in both A/P compartments. (**b**) Localization of Arm in an *en*>*IRmfl* silenced disc (v46282 line). *mfl* silencing causes a strong reduction of Arm protein, with Z-stack analysis (right panels) demonstrating loss of apical localization (right panel). In **a** and **b**, green dots mark the boundary between the A and P compartments within the enlargements; red dots mark XZ projection enlargements. (**c** and **d**) Confocal analysis of wing discs stained with phalloidin. (**c**) In *en*>GFP control discs, F-actin concentrates in two stripes of cells adjacent to the D/V boundary. (**d**) In *en*>GFP, *en*>*IRmfl* silenced discs (v46282 line in the picture), F-actin accumulation is strongly reduced within the whole silenced domain, and particularly at the D/V margin. GFP is in green and phalloidin in red or gray. Green dots mark the A/P boundary. a, Apical; b, basal

The Arm protein is a known effector of Wg signaling and has a dual role: as a component of the cell adherens junctions on the one hand and as a nuclear transcription factor transducing Wg signal on the other hand.^35^ Within the wing disc, Arm concentrates apically, in which it binds transmembrane cadherins to build up the adherens junctions that connect actin filaments across polarized epithelial cells^36^ (see Figure 6a). Interestingly, upon *mfl* silencing directed by *en*-Gal4, Arm accumulation was strongly reduced in the whole silenced area, and most evidently at the D/V boundary. Moreover, confocal Z-stack analysis revealed a reduction of apical Arm accumulation, so that cells had lost their polarity (Figure 6b). Identical results where obtained when silencing was directed by the *omb*-Gal4 driver (Supplementary Figure 4). Reduction of apical Arm is consistent with the observation that Wg overexpression in the wing discs induces a transient reduction of membrane-associated Arm, this way allowing an immediate decline of cell adhesion that facilitates structural reorganization of the cytoskeleton.^37^ Similarly to Arm, F-actin accumulation was also heavily reduced, and accumulation at the D/V boundary disrupted (Figures 6c and d and Supplementary Videos 2 and 3). Hence, the concomitant induction of cell death and proliferation is accompanied by extensive cytoskeletal remodeling. The involvement of pseudouridine synthase activity in cytoskeletal dynamics and cell adhesion is worth noting, and it has previously been described also in human cells.^38^

**Figure 7:**
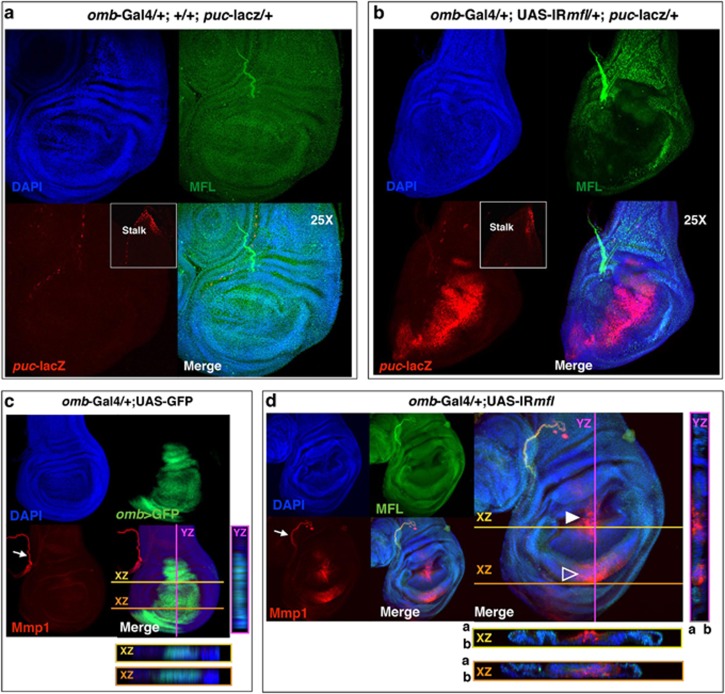
Mfl depletion induces JNK and Mmp1 ectopic activation. Confocal analysis of wing discs collected at 120 h AED. (**a**) In *omb*>*puc-lacZ* control discs, activation of the JNK pathway, marked by the *puc-lacZ* reporter, is restricted to the stalk cells (inset) and to some dispersed cells.^47^ (**b**) In *omb*>*IRmfl*; *puc-lacZ* silenced discs (v46282 line in the picture), JNK is ectopically and strongly activated. In (**a** and **b**), DAPI (4',6-diamidino-2-phenylindole) is in blue, Mfl in green and *β*-gal in red. (**c**) Mmp1 expression in *omb*>GFP control discs marks the trachea (arrow) and the stalk cells (not in frame), where it overlaps *puc-lacZ* expression. Z-stack analysis (see XZ and YZ projections) confirms Mmp1 absence in the *omb* domain. (**d**) In *omb*>*IRmfl* silenced discs (v46282 line in the picture), Mmp1is strongly and ectopically induced in the MFL-depleted domain; in the D compartment, Mmp1 enrichment flanks the A/P border (closed arrowhead) within the area of JNK activation (compared with **b**), whereas in the V compartment, Mmp1 concentrates in a small domain (open arrowhead) overlapping the Wg-expressing inner ring. In both areas, Z-stack analysis (see XZ and YZ projections) confirms Mmp1 active secretion within the silenced domain. DAPI is in blue; GFP (**c**) and Mfl (**d**) in green and Mmp1 in red. a, Apical; b, basal

Given that the JNK pathway is known to be involved in cytoskeletal remodeling during both apoptotic^39, 40^ and regenerative processes,^41, 42, 43, 44, 45^ we checked whether it was specifically induced upon Mfl depletion. We then followed the expression of *puckered* (*puc*), a JNK downstream effector,^46^ taking advantage of the widely used *puc*-*lacz* reporter. As described,^47^ *puc*-*lacz* expression in wild-type discs is restricted to the stalk region, where wing discs are connected to the larval epidermis (Figure 7a). In contrast, expression of this reporter was found strongly induced within the silenced discs (Figure 7b). Along the A/P border, JNK ectopic induction matched the local clusters of pyknotic nuclei, suggesting that it resulted in a local cell death. However, in the ventral regions it overlapped the areas of Wg accumulation, suggesting that in these regions JNK activity could instead promote proliferation, in keeping with the dual role recently suggested for this pathway.^45^ JNK also has a well conserved role in the induction of Mmps that degrade the extracellular matrix and are strongly expressed during regeneration.^48, 49^ Specifically, *Drosophila* Mmp1 is directly involved in re-epithelialization after wound healing, remodeling of the basement membrane and cytoskeletal reorganization.^50, 51^ Not surprisingly, Mmp1 was strongly induced along the A/P border and ventrally, where it matched the area of Wg overexpression at the middle of the inner ring (Figures 7c and d).

### Mfl depletion promotes EMT in a cell-nonautonomous manner

**Figure 8:**
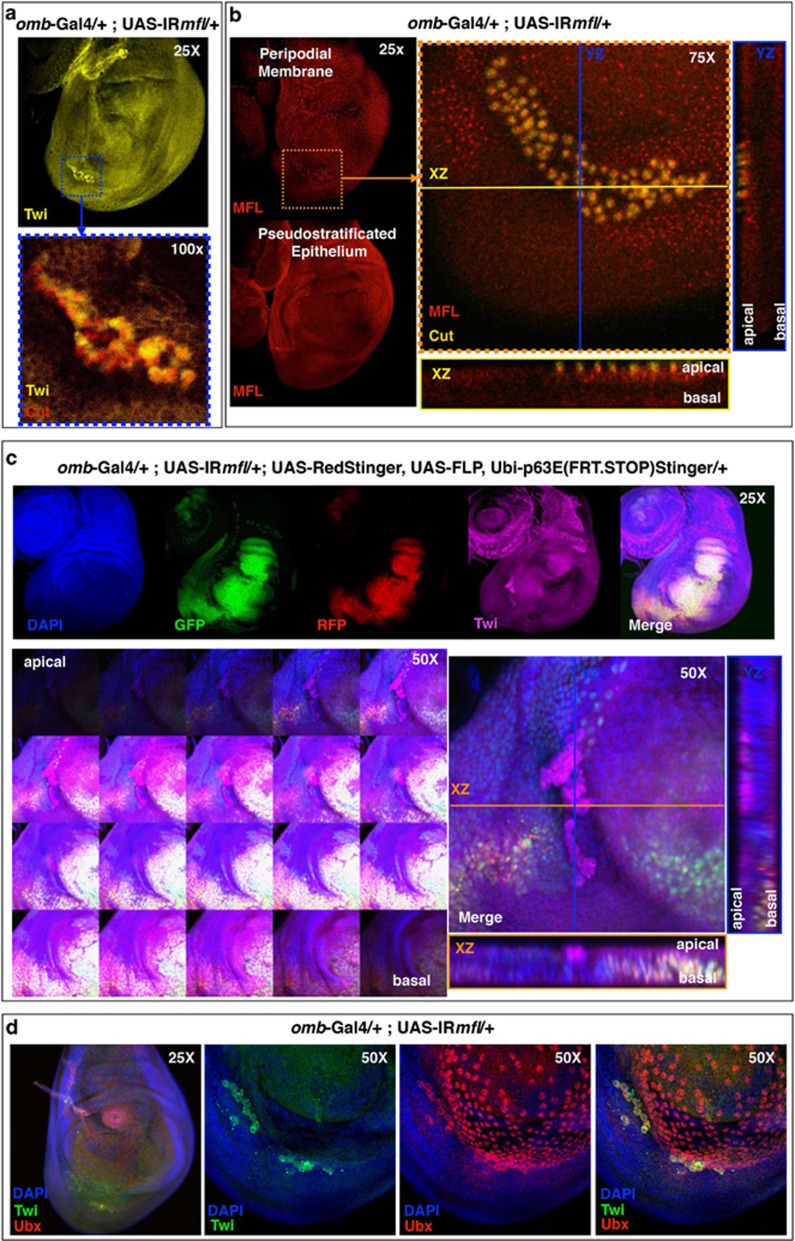
Mfl depletion promotes EMT in the contiguous peripodial membrane. Confocal analysis of wing discs collected at 120 h AED. (**a**) An *omb*>*IRmfl* silenced disc (v46282 line) costained with anti-Twist (on the top) and anti-Cut antibodies, showing an islet of double positive cells (enlargement at the bottom; Twist is in yellow and Cut in red). (**b**) Z-stack images captured at different planes show that the Twist-Cut-positive islet lies apically, in the peripodial membrane. As this membrane is not included in the *omb* silenced domain, these cells were actively accumulating the Mfl protein within their nucleoli; conversely, cells of the underlying silenced pseudostratified epithelium are efficiently depleted (Mfl in red and Cut in yellow). (**c**) G-TRACE experiments in which the *omb* silenced domain was labeled by GFP and RFP expressed by UAS-Stinger vectors and stained with DAPI (4',6-diamidino-2-phenylindole) and Twist antibody (top panel). In the middle, Z-stack images captured at different focal planes ( left) and orthogonal projections (right). Note that Twist-positive cells (in magenta) remain fully unstained by both GFP and RFP and lie apically, within the peripodial membrane. DAPI is in blue, GFP in green, RFP in red and Twist in magenta. (**d**) Twist-positive cells faintly express also Ubx, a typical marker of the peripodial membrane, indicating that they are in a state of fate transition. DAPI is in blue, Twist in green and Ubx in red. Twi, Twist

The concomitant occurrence of Wg overexpression, reduction of adherens junctions, loss of apicobasal polarity, JNK and Mmp1 induction raised the intriguing possibility that Mfl depletion could also trigger changes in cell fate, and possibly give rise to EMT. EMT occurs in many developmental events but, if induced by pathological conditions, can lead to tumourigenesis.^52^ As X-DC is characterized by a still unexplained susceptibility to malignancy, the possibility that pseudouridine synthase depletion could trigger EMT was of great interest. We thus stained the silenced discs with an antibody directed against Twist, a typical marker for mesenchymal cells known to be involved in EMT.^53, 54^ Twist is expressed in presumptive mesodermal cells and not in epithelia,^55^ and thus no disc cell was expected to be positively stained. Intriguingly, patches of Twist-positive cells were instead detected in about 95% of the silenced discs (Figure 8a). These cells also ectopically expressed Cut, an additional marker of myoblasts,^55, 56^ further confirming the acquisition of mesoderm identity and the occurrence of EMT. Surprisingly, Z-stack confocal analysis lead to locate these myoblasts within the peripodial membrane, that is, the overlying squamous epithelium that lies outside the *omb* expression domain and thus was not silenced (Figure 8b). Note that, as awaited, Cut-positive cells are never present in the peripodial membrane of control discs (Supplementary Figure 5). We then wondered whether the observed myoblasts originated from the underlying silenced epithelium and then migrated above or, alternatively, directly arose from the unsilenced tissue. To investigate this aspect, we performed a lineage-tracing experiments by using the G-TRACE system, which is based on the expression of a pair of GFP-RFP Stinger reporters.^57^ In this system, cells that had a previous Gal4-dependent activation of the GFP reporter, even if transient, remain GFP-labeled; conversely, cells showing only an active real-time expression of the Stinger vector become RFP-labeled. In our experiments, Twist/Cut-positive cells were never GFP- or RFP-labeled, clearly indicating that they were not expressing the *omb*-Gal4 driver nor activated it at any previous developmental stage (Figure 8c). This result confirmed that these cells derive from the peripodial membrane. To learn more, we used an anti-Ubx antibody that marks the majority of peripodial cells.^58, 59^ Intriguingly, the Twist/Cut-positive cells faintly expressed also Ubx, indicating that they are in a state of cell fate transition (Figure 8d). This result ruled out also the possibility that these myoblasts could derive from the adepithelial cells abutting the wing disc in the notum region, as those myoblasts do not express Ubx.^58^ EMT in the peripodial membrane was identically induced in a different *mfl* silencing line (no. 36595; Supplementary Figure 6), confirming the occurrence of cell-nonautonomous fate changes.

## Discussion

In *Drosophila* wing discs, cell death provoked by a variety of approaches, including disc transplantation, exogenous injuries or localized induction of proapoptotic genes, can induce regenerative growth.^60^ In response to death, neighboring cells are stimulated to proliferate and reconstitute tissue loss, a process defined as apoptosis-induced proliferation.^28, 61, 62^ Here we show that the level of expression of pseudouridine synthase is crucial for tissue homeostasis and that its local reduction triggers apoptosis-induced proliferation, as typically observed during regeneration.^63^ This regenerative stimulus is enhanced by blocking the execution of death by p35 expression, which, as occurring after several different types of tissue injuries, elicits hyperplastic overgrowth.^64^ As both apoptotic and proliferating cells are identically Mfl-depleted, our results outlined an unexpected context-dependent effect of pseudouridine synthase level. Possibly, as a consequence of their differentiation status, different cell sub-populations respond in a reverse manner to lessening of this enzyme: those more susceptible undergo apoptosis, whereas others hyperproliferate, acting as a blastema.^29^ This dual effect also establishes for the first time that pseudouridine synthase depletion *per se* does not hamper proliferation, as generally considered; on the contrary, under mitogenic stimuli the depleted cells are able to overproliferate vigorously. This finding further supports the view that this enzyme has a more specialized than a general effect on ribosome functionality.

As described in regenerative processes, massive epithelial remodeling occurs in the silenced tissue. Loss of Arm apical localization, reduction of F-actin polymerization and JNK and Mmp1 ectopic activation, altogether converged to underscoring cytoskeletal rearrangement dynamics and massive epithelium reorganization. In our experiments, the sustainment of the proliferative activity correlates with the activation of Wg and JAK-STAT mitogenic pathways, both of which are found ectopically induced in the depleted areas. However, the Wg signal was actively produced not only by the apoptotic but also by surrounding cells, where it partially overlapped with JAK-STAT ectopic induction. These features suggest the involvement of long-range intercellular signaling in response to pseudouridine synthase depletion. Indeed, an interesting conclusion that can be drawn by our experiments is that cell–cell communication is likely to have a key role in the pseudoridine synthase loss-of-function phenotype. This conclusion is further supported by the striking discovery that depletion of *Drosphila* dyskerin can trigger cell fate changes in a cell-nonautonomous manner. Unexpectedly, in response to Mfl silencing, EMT occurs in the adjacent unsilenced peripodial membrane, where groups of cells exhibited ectopic expression of myoblast markers. Considering the pivotal role played by EMT in development, regeneration and stem cell behavior,^65^ this result is of utmost importance. Moreover, it emphasizes the important role of dyskerin, which is also reflected by extensive consequences of its depletion on many processes during fly development.^17^ Indeed, the regenerative growth described here is likely to reflect a general intrinsic homeostatic mechanism occurring in the context of physiological metabolic perturbance. In developing tissues, a local decrease in the amount of pseudouridine synthase, or in its activity, can occur stochastically or be caused by developmentally or metabolically regulated processes. Our results show that such variations can act not only as an apoptotic trigger but also as a regenerative stimulus, and thus nicely fit with the general observation that a tight control of the level of this enzyme is crucial in human cultured cells^66^ and developing organisms as well.^4, 17^

Collectively, the effects observed upon Mfl depletion all converge to make the tumor predisposition observed in the X-DC patients^67^ much easier to understand. Indeed, considering the high degree of conservation of all the pathways affected by Mfl reduction, we foresee that the core wiring diagram of pseudouridine synthase tasks might be conserved between flies and mammals. We surmise that *in vivo* experimental approaches in this animal model may help to better define the wide range of biological processes that interlace with these multifunctional proteins, and that perturbance of cell–cell interactions, so far largely ignored in the studies of X-DC pathogenesis, may represent a relevant aspect of the disease.

## Materials and Methods

### Fly stocks

Flies were raised on standard *Drosophila* medium at 25 °C. The *en*-Gal4, *omb*-Gal4/FM7, UAS-GFP/CyO, 10XSTAT92E-dGFP/TM6C, *puc-lacZ*/TM3Sb, UAS-2XEGFP, UAS-RedStinger, UAS-FLP, Ubi-p63E(FRT.STOP)Stinger and UAS-*IRmfl* (no. 36595) strains were obtained from the Bloomington Drosophila Stock Center at Indiana University (BDSC, Bloomington, IN, USA); UAS-*IRmfl* RNAi (v46282) was obtained from the Vienna Drosophila RNAi Center (VDRC, Vienna, Austria). *wg-lacZ*/CyO and UAS-*p35*/TM3Sb stocks were kindly provided by L Johnston (Columbia University, New York, NY, USA).

### Mounting adult wings

Wings were removed from adult flies, dehydrated in 100% ethanol for 5 min and placed on a microscope slide to allow ethanol to evaporate. A small drop of Euparal Mounting Medium (Roth, Karlsruhe, Germany) was dropped onto the wing and a glass coverslip placed on top. Images were captured with a Spot digital camera and a Nikon E1000 microscope (Nikon Instruments Europe, Tokyo, Japan).

### Immunofluorescence stainings

Wing discs were dissected, fixed and immunostained as described in Tortoriello *et al.*^17^ Antibodies used were as follows: customer rabbit polyclon alantibody against Mfl (Sigma-Aldrich Inc., St. Louis, MO, USA; dilution 1 : 100); mouse monoclonal antibodies against Wingless, Cut, Arm, Mmp1, *β*-galactosidase, ultrabithorax (Ubx; Hybridoma Bank, University of Iowa, Iowa City, IA, USA; dilution 1 : 50 anti-Wg, 1 : 100 anti-Cut; 1 : 50 anti-Arm, 1 : 50 anti-Mmp1 mixture of 5H7B11, 3B8D12 and 3A6B4, 1 : 250 anti-*β*-Gal and 1 : 50 anti-Ubx); rabbit polyclonalantibody against Twist (Yin *et al.*;^68^ dilution 1 : 50; gift from M Frasch); rabbit polyclonal antibodies against pH3 and cleaved Cas3 (Cell Signaling Technology, Danvers, MA, USA; dilutions 1 : 100 anti-pH3 and 1:500 anti-Cas3). Fluorescent secondary antibodies were from Jackson ImmunoResearch (Dianova, Hamburg, Germany) and used at a final dilution of 1:200. Rhodamine phalloidin conjugate for actin cytoskeleton staining were obtained from Molecular Probes (Eugene, OR, USA; dilution 1 : 250). Confocal images were obtained with a Bio-Rad MRC1024 (Bio-Rad, Munich, Germany) or Zeiss LSM510 (Carl Zeiss, Jena, Germany) confocal microscope.

### Labeling of S-phase cells

For EdU immunohistochemistry, the Click-iT EdU Imaging Kit (Invitrogen, Carlsbad, CA, USA) was used. Discs were dissected and incubated in 10 *μ*M EdU in Ringer's for 20 min or 2 h and, following EdU labeling, fixed and immunostained as described in Tortoriello *et al.*^17^ Afterwards, they were incubated in 1x Click-iT reaction cocktail for 30 min, washed thoroughly in PBS and mounted.

### Z-stack analysis

All captured pictures (in RAW format) have been analyzed and processed with ImageJ v1.440 software (National Institutes of Health, Bethesda, MD, USA). Z-stack analysis was performed by using STACK>ZProjection and STACK>Orthogonal views ImageJ plug-in.

## Abbreviations

H/ACA snoRNP: H/ACA box small nucleolar ribonucleoprotein
snoRNA: small nucleolar RNA
X-DC: X-linked dyskeratosis congenita
Omb: optomoter blind
En: engrailed
Dpp: decepentaplegic
Wg: Wingless
Arm: Armadillo/*β*-catenin
Cas3: caspase-3
pH3: phosphohistone H3
EdU: 5-ethynyl-2′-deoxyuridine
JAK-STAT: Janus kinase-Signal Transducer and Activator of Transcription
JNK: c-Jun N-terminal kinase
Puc: puckered
Mmp1: matrix metalloproteinase-1
Ubx: ultrabithorax
EMT: epithelial–mesenchimal transition
