# Supplementary figures and images for: Loss of Drosophila pseudouridine synthase triggers apoptosis-induced proliferation and promotes cell-nonautonomous EMT
Source: Cell Death Dis. 2015 Mar 26;6(3):e1705–. doi: 10.1038/cddis.2015.68 (PMC4385944; doi:10.1038/cddis.2015.68)

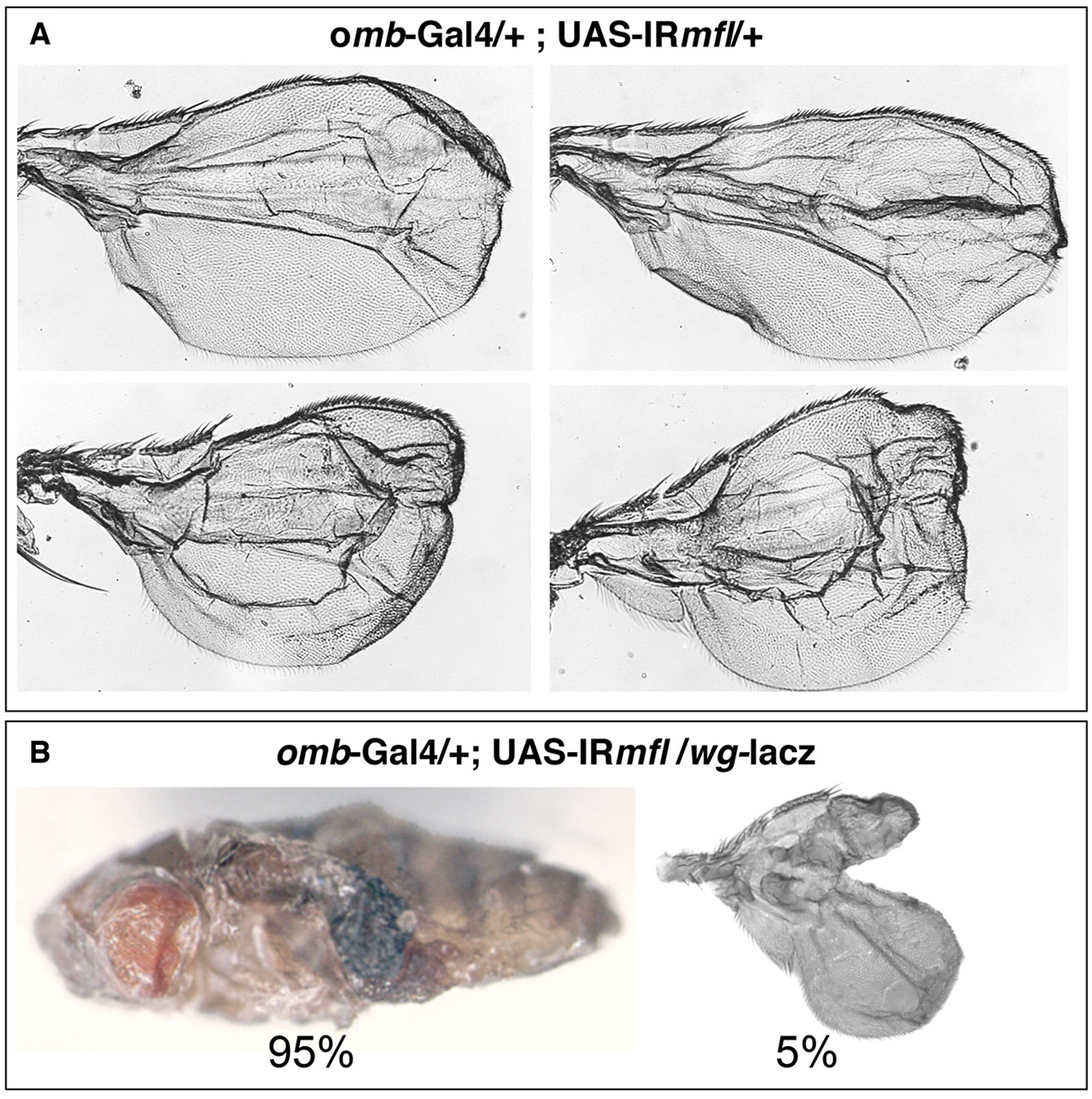

Supplement: Supplementary Figure 1 [file cddis201568x1.tif]

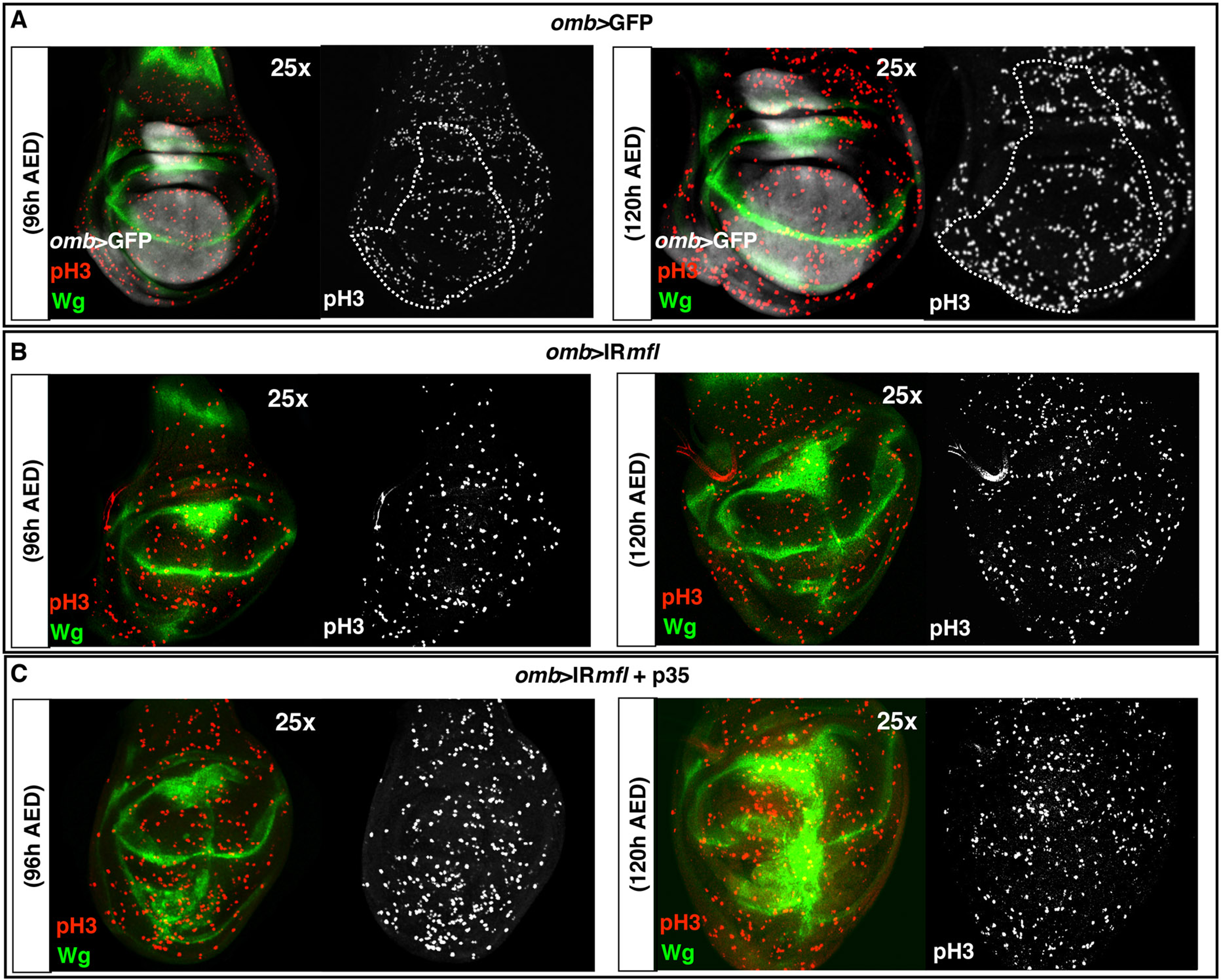

Supplement: Supplementary Figure 2 [file cddis201568x2.tif]

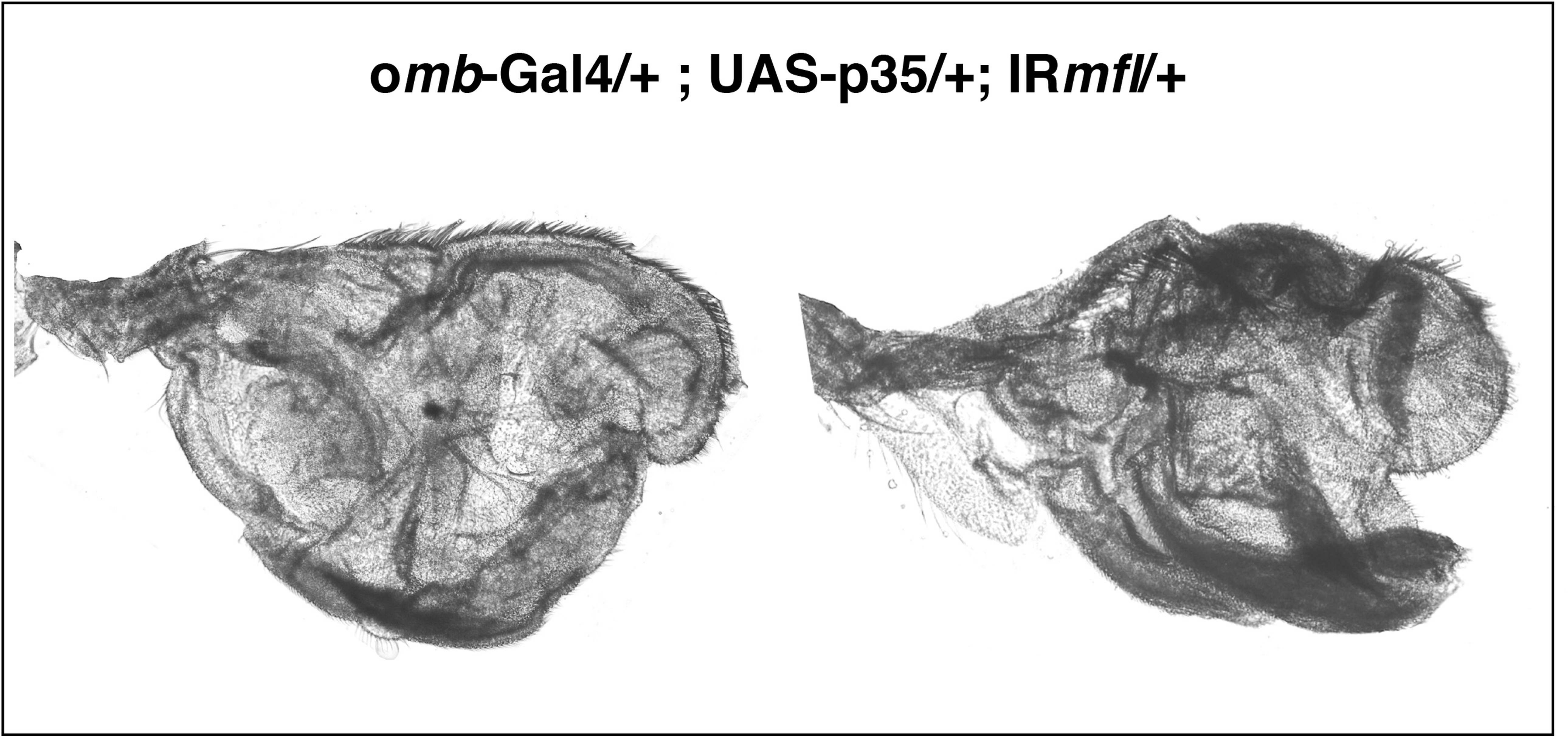

Supplement: Supplementary Figure 3 [file cddis201568x3.tif]

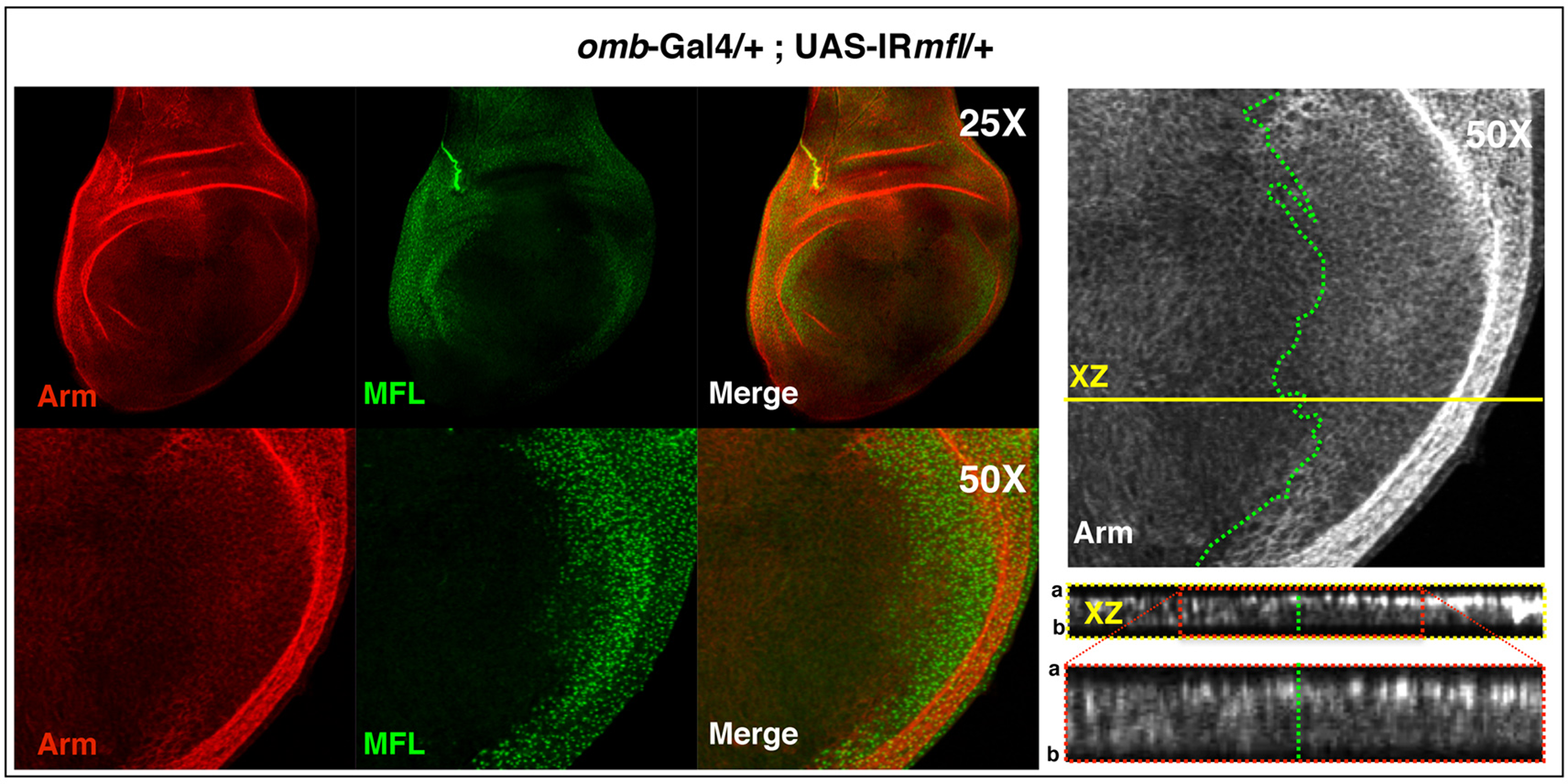

Supplement: Supplementary Figure 4 [file cddis201568x4.tif]

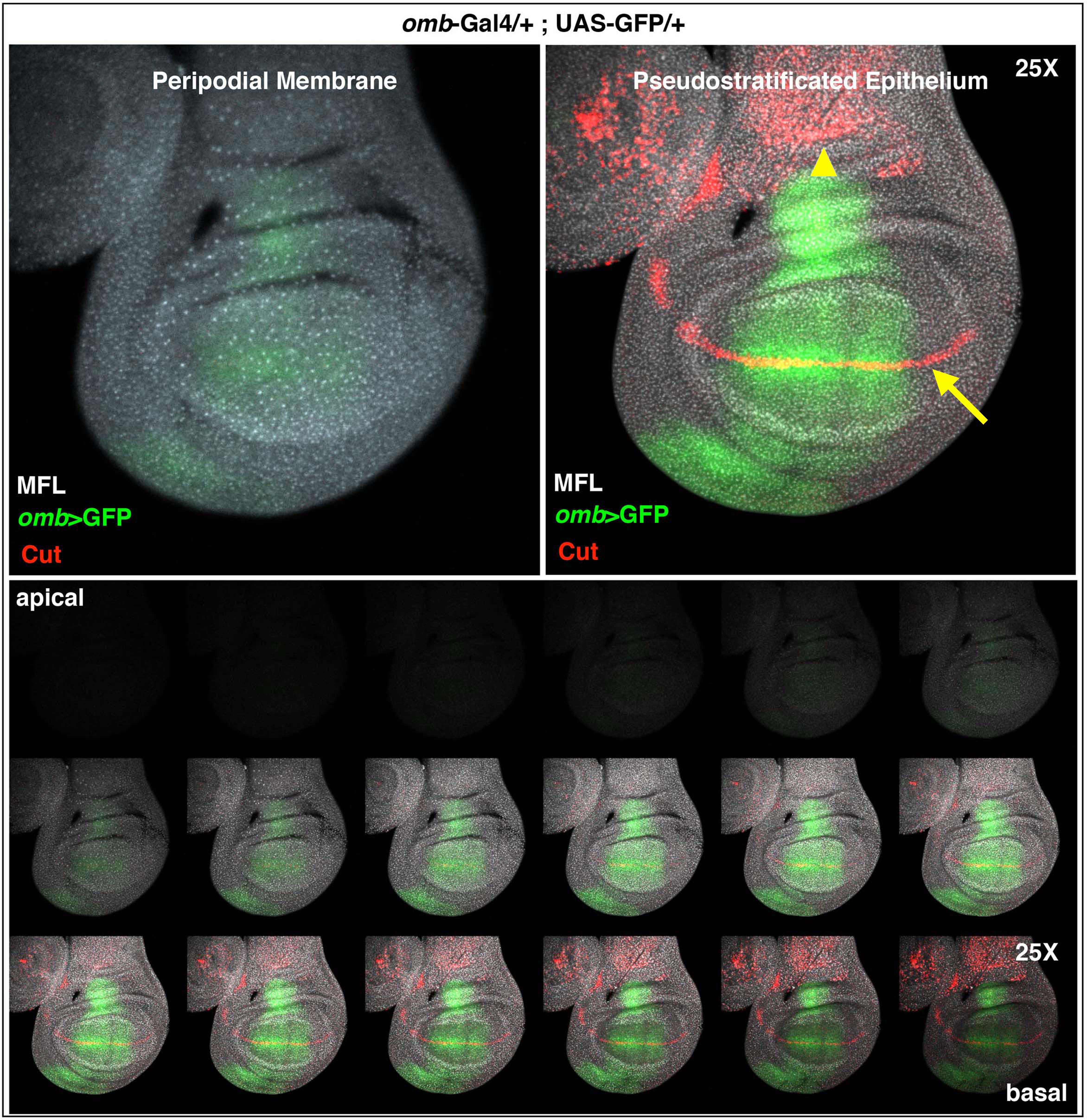

Supplement: Supplementary Figure 5 [file cddis201568x5.tif]

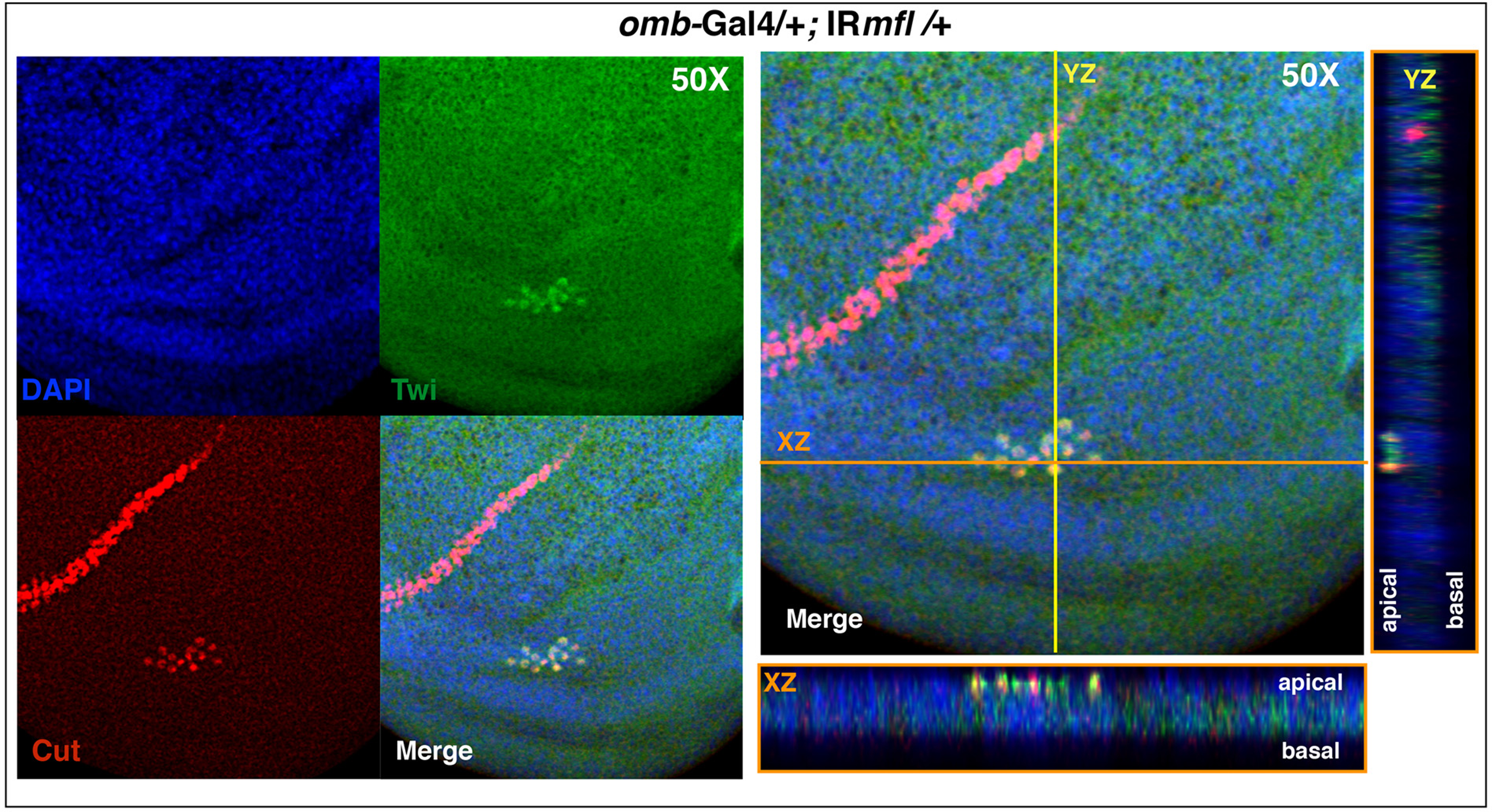

Supplement: Supplementary Figure 6 [file cddis201568x6.tif]
