# Supplementary material for: Loss of Drosophila pseudouridine synthase triggers apoptosis-induced proliferation and promotes cell-nonautonomous EMT
Source: Cell Death Dis. 2015 Mar 26;6(3):e1705–. doi: 10.1038/cddis.2015.68 (PMC4385944; doi:10.1038/cddis.2015.68)
Supplement: Supplementary Information [file cddis201568x10.doc]

**Supplementary informations**

**Supplementary Figure 1**

***wg* haploinsufficiency worsens the silenced phenotype.**

**A.** Phenotypic variability of *omb*>IR*mfl* adult wings. Wing morphology is always severely affected and invariantly accompanied by blistering. **B.** Heterozygosity for the *wg*-*lacz* loss of function allele lead to 95% of mortality at the pupal stage, while rare adult escapers (5%) display underdeveloped wings that miss the *omb* domain.

**Supplementary Figure 2**

**Mitotic activity within the Mfl depleted domain.**

Confocal analysis of wing discs at 96 h and 120 h AED. **A**. An *omb*>GFP control disc stained with pH3 antibody to mark mitotic cells. **B**. In *omb*>IR*mfl* silenced discs (#36595 line in the picture), the overall proliferation rate was not reduced, despite the occurrence of massive apoptosis (see Figure 1); pH3-positive cells were detected within and around the Wg-secreting areas. **C.** In *omb*>IRmfl, UAS-p35 discs (#36595 line in the picture), pH3 staining reveals a further increase of mitotic activity. GFP is in gray (the dotted line encircles the *omb* domain); pH3 in red or gray; Wg in green.

**Supplementary Figure 3**

**Hyperplastic phenotype of wings displayed by *omb*-Gal4/+; UAS-IR*mfl*/+; UAS-p35/+ adult escapers.**

In presence of p35, most silenced pupae do not eclose; rare adult escapers exhibit wings with hyperplastic phenotype.

**Supplementary Figure 4**

**Mfl silencing reduces apical Arm accumulation**

Localization of Arm protein in *omb*>IR*mfl* silenced discs (v46282 line). In keeping with data obtained upon *mfl* silencing directed by *en*-Gal4, Arm protein is reduced within the *omb* silenced domain, and Z-stack analysis (right panels) shows that the apical localization is disrupted (right panel; green dots on the top and red dots on the bottom enlargement mark the boundary between the silenced and unsilenced domains). Arm is in red or gray; MFL is in green.

**Supplementary Figure 5**

**No Cut positive cell is present in the peripodial membrane of control discs.**

Confocal analysis of an *omb>*GFP control disc at 120 h AED, costained with Cut and Mfl antibodies. On the left, uppermost planes focused on the peripodial membrane; on the right, basal planes focused on the pseudostratified epithelium. On the bottom, all captured focal planes, from apical to basal. Note that Cut positive cells are absent at the level of the peripodial membrane, while in the same disc they are clearly visualized at D/V boundary of the underneath pseudostratified epithelium (arrow) and in correspondence of the adepithelial cells in the notum region (arrowhead). Mfl is in gray, Cut in red, GFP in green.

**Supplementary Figure 6**

**EMT in the peripodial membrane is induced by different *mfl* silencing transgenes.**

Z-stack analysis of *omb*>IR*mfl* silenced discs (#36595 line) co-stained with anti-Cut and anti-Twi. As observed for the v46282 line, Cut/Twi positive cells are detectable in the peripodial membrane of silenced discs. Dapi is in blue, Cut in red and Twi in green.

**Supplementary Video 1. Epithelial remodeling in Mfl depleted discs.**

Z-stack analysis of an *omb*>IR*mfl* silenced disc (v46282 line). Upon Mfl depletion, the disc tissue appeared crumpled and folded, indicating the occurrence of extensive reorganization of the silenced epithelium. Mfl is in red; Wg in blue. The dotted line encircling the silenced disc outlines its bent shape.

**Supplementary Video 2**

**F-actin accumulation in an *en*-Gal4/+ control disc.**

Phalloidin staining and Z-stack analysis highlights F-actin concentration in the two stripes of cells adjacent to the D/V boundary.

**Supplementary Video 3**

**Mfl depletion reduces F-actin accumulation**

Z-stack analysis of an *en>*IR-*mfl* silenced disc (v46282 line in the video). Upon *mfl* silencing, Note that F-actin accumulation was reduced, mainly at the D/V boundary.
